# Teratogenic Genesis in Fetal Malformations

**DOI:** 10.7759/cureus.13149

**Published:** 2021-02-05

**Authors:** Roohi Afshan Kaleelullah, Neha Garugula

**Affiliations:** 1 Dentistry, California Institute of Behavioral Neurosciences & Psychology, Fairfield, USA; 2 Dentistry, Dr. Nandamuri Taraka Rama Rao University of Health Sciences, Virginia, USA

**Keywords:** birth defects, teratogenic fetal abnormalities, prenatal risks, congenital disorders, in-utero complications, pregnancy risk factors, teratology, fetal syndromes, non-genetic, precautions

## Abstract

Congenital anomalies can occur during the developmental stages of the embryo, from abnormal genetics passed on from the parents or from vivid environmental factors. While advanced technologies are able to detect chromosomal abnormalities, there are many unknown non-genetic variants. Teratogenic factors pose a greater risk to the fetus, as these abnormalities may go undetected until birth. These malformations are the origin of the infant's postnatal illness and disability. The defects can also lead to mortality. The loss can also affect families, as they are affected by not only the loss but also financially. Most of the teratogenic-induced anomalies, once detected, maybe rehabilitated naturally. Those who do require medical intervention pose their own risks, similar to those of infections. Therefore, environmental exposure to teratogens can create long-lasting effects that range from infertility, intrauterine growth restriction, structural defects, and functional central nervous system abnormalities that may lead to fetal death.

## Introduction and background

History

Environmental factors work as teratogens to cause congenital disorders in a developing fetus. The chromosomes and genes of fetuses and children with congenital disorders caused by a teratogen are genetically normal. While multifactorial congenital disorders stem from various sources, including obvious genetic factors, teratogens are solely triggered through conditions outside of the womb such as drugs, chemicals, or infections. As congenital disabilities are detected, medical professionals must consider multifactorial congenital disorders while keeping in mind that most congenital disabilities stem from teratogens [1-2].

For the first time, teratology was discovered in the 1930s after various experiments were performed on pregnant pigs. In these experiments, pigs were fed with a vitamin A deficiency diet. Eventually, all those piglets experienced devastating malformations, the principal loss of eyes [1]. Sir Norman Gregg, in 1941 came across the human rubella virus as the first most human teratogen. Exposure to this virus in utero led the way for heart defects and congenital cataracts [2]. As science developed, the effect of xenobiotic agents on embryos was demonstrated by experimenting on animals with congeners of biologically predominant molecules, likely amino acid analog azaserine. In the 1950s, aminopterin was used to terminate the pregnancy. Instead, several disabled newborns were born after the drug failed to abort the pregnancies [3].

Lack of public health care for pregnant women is the main contributor to contact with teratogenic variants. Women with a lack of medical care are more likely to engage in alcohol consumption and drug abuse. These are also typically the same factors; the progeny of socially disadvantaged women is more vulnerable to newborn defects [4].

## Review

Teratogenic factors and their risks

Alcohol

During the primary four weeks of pregnancy, the baby develops the foremost crucial organs and organ systems in their body, such as the heart, central nervous system, eyes, arms, and legs. The baby's brain starts developing around the third week of its intrauterine life and steadily matures together with the pregnancy. Whenever the mother consumes alcohol, it can easily bypass through the placenta, giving rise to irrevocable changes. Alcohol breakdown within the baby’s body is way slower than the fully mature adult body. Thus, causing irreparable changes in fetal development. One can barricade these changes by avoiding alcohol consumption during pregnancy and protecting the baby from fetal alcohol spectrum disorder [5].

Retinoic Acid

Vitamin A is a vital vitamin that helps to differentiate the cellular epithelium. The Surplus amount of fat-soluble vitamins and retinoids of pregnant women often end up in fetal malformations. The involved organs are the fetal skull, face, limbs, eyes, central nervous system due to excess retinoids. Using 13-cis retinoic acid by pregnant women may end up in developmental anomalies during prenatal and postnatal life of the infants, like cleft palate, anotia or microtia, heart defects, and thymus abnormalities. Avoiding the retinoids' superfluity during pregnancy can make the baby sleep in this world as an entire human with no defects [6].

Antibiotics

Many antibiotics are recognized to be teratogenic and must be avoided completely during pregnancy. Those are streptomycin, kanamycin, and tetracycline, which interrupt fetal development. These drugs can cause hearing impairment, hypoplasia, and tarnishing of long bones and teeth, respectively. Even more, broad-spectrum antibiotics can drag out the latency period. Narrow-spectrum antibiotics are considered to be safer than broad-spectrum antibiotics. To be on the safe side, every pregnant woman can contemplate the subsequent. Usage of antibiotics as long as prescribed by a specific practitioner or if necessary, to be taken. Avoiding antibiotics during the first trimester of pregnancy because the major fetal structural transformation occurs during this term. Thus, divert the risk of iatrogenic exposure. Using a low dose of antibiotics. Limiting the use of over-the-counter drugs will avoid the interlinking of the chemical properties of medicine prescribed versus self-instructed over-the-counter drugs [7].

The effects of antibiotics in fetal malformations are mentioned respective to different types of antibiotics are as follows [8]:

**Table 1:** Effects of antibiotics [8]

Clindamycin	Major congenital malformations including musculoskeletal system anomalies and ventricular/atrial septal defect
Doxycycline	Increased risk for cardiac malformations and ventricular/atrial septal defect
Erythromycin	Nephrotic system malformations
Macrolides	Digestive system disorders
Moxifloxacin	Respiratory abnormalities
Ofloxacin	Major congenital malformations
Phenoxymethylpenicillin	Nervous system malformations
Quinoline	Urinary system abnormality
Tetracycline	Permanent discoloration of a child’s teeth and bone disorders

Anticancer drugs

For several years, the number of women diagnosed with cancer has increased in huge numbers during pregnancy. The most frequent cancers reported in pregnancy are breast cancer, cervical cancer, malignant melanoma, and malignant lymphoma. Congenital malformations are more likely to occur if the cytotoxic anticancer drugs are prescribed during the first trimester of pregnancy because the fetus at this stage encounters rapid cell division and organogenesis. Antineoplastic treatment provided to a pregnant lady at this time may involve fetal risks, such as stunting growth, abnormalities, systemic toxicity, abortion, and death. Several other effects likely to occur are immunosuppression, adrenal insufficiency, and hematopoietic depression. Thus, their use should be avoided unless the mother's life and health are compromised [9].

Even though chemotherapy is administered during the second trimester onwards, there is no escape from the effects of fetal malformations. The risks include intrauterine growth retardation, an increase in premature labor, and premature rupture of membranes. For these reasons, fetal tracking throughout the pregnancy during each chemotherapy cycle is mandatory. However, early delivery of the infant is recommended when the mother can be treated adequately. Pregnant women who are freshly recovered from any chemotherapeutic treatment should not breast-feed their infants [10].

Warfarin

Coumadin is totally absorbed from the small intestine, attached loosely to albumin in the blood, and degenerated within the liver. Warfarin is the competitive inhibitor of vitamin K. With the consumption of Coumadin, all the clotting factors (II, VII, IX, X) impede their activity; thus, anticoagulation takes place [11].

Warfarin can easily cross the placental barrier and enter the fetal bloodstream. It has a lower molecular weight, causing fetal warfarin syndrome-children with fetal warfarin syndrome exhibit numerous otolaryngological anomalies, together with atypical bone growth. Imbibing warfarin during the first trimester of pregnancy can cause physical abnormalities like nasal hypoplasia and bone stippling. After the first year of life, the stippled areas can be reabsorbed. Warfarin taken during the second and third trimester causes central nervous system anomalies. They are mental retardation, optic atrophy, delayed development, seizures, and microcephaly. Consuming higher doses of warfarin greater than 5mg daily can immediately result in fetal death or stillbirth. Warfarin syndrome can be prevented by not prescribing the warfarin drug to the women trying to conceive and avoid it before five days of conception, as the drug can stay in the mother’s body for up to five days. For women having prosthetic heart valves, anticoagulation medicine cannot be suspended. Instead, they can go with alternate anti-coagulants which cannot cross the placental barrier, like ximelagatran [12].

Bodily and Facial Anomalies with Warfarin

Complete skeletal abnormalities are stereotyped in fetal warfarin syndrome. Generalized bone depletion causes rhizomelia, inordinate short limbs (brachydactyly), short fingers and toes, shorter neck, scoliosis, and stippled epiphyses pectus carinatum, and the sunken sternum frequently represents the fetal warfarin syndrome. Nasal hypoplasia, stippling of bones, microcephaly, blindness, delayed facial development, absence of nasal septum, microphthalmia, telecanthus, ectopic lacrimal duct, choanal atresia, the narrow airway at the posterior nasal cavity, respiratory distress, cleft lip, laryngomalacia, and large soft protrusions into larynx [12-13].

Infectious agents

Rubella

The rubella virus affects more than 100,000 children each year. The reservoir for this virus is humans. It is transmitted through respiratory droplets. After entering the human tract, the virus flows into the mucosa or the local lymph nodes and replicates. After that, it slowly enters the bloodstream and spreads to the regional lymph nodes. This is the stage of the second viremia. The virus incubation period is 14 days, and it spreads from the respiratory droplets to acquire the other human host and starts its cycle again [14]. 

The virus mainly attacks infants with congenital rubella syndrome. This illness results from maternal infection with rubella during pregnancy. The anomalies are more common in the fetus when the mother gets the rubella infection during her first trimester. The virus can easily travel through the placenta and infects the major organ systems of the infant. During early pregnancy, the fetus's infection results in miscarriages, stillbirths, or any severe abnormality affecting the infant. The percentage of risk factors reduces if the mother is 20 weeks pregnant or even more with the rubella infection [14-15].

Congenital rubella syndrome's common defects are* c*ataracts, congenital heart disease, hearing impairment, developmental delay, glaucoma, retinitis, microcephaly, mental retardation, bone disease, diabetes. Infants with congenital rubella syndrome can be with multiple defects or a single defect. The most common solo defect is hearing impairment. Congenital rubella syndrome can be eradicated by protecting the mother and infant by giving MMR (mumps, measles, and rubella vaccine) [14-15].

Mumps

Mumps is a viral condition that mainly affects humans. This virus's transmission is through direct contact or the respiratory droplets of a person already infected with this virus to the fresh candidate. After the virus sets in the human body, it rapidly moves to the respiratory system, where it can replicate. Its incubation period is 16-18 days. The infectious period is two to five days. Mumps' onset can be recognized by pain, tenderness, as well as swelling in one or both parotid salivary glands. The side of the swollen area pushes the angle of the ear to be outwards [16]. When occurring in pregnant women, mumps is not as severe as other potential infections. However, like any other infections, Mumps can cause illness in infants if the mother contracted the virus during her early pregnancy stages. If the virus infects the mother in her first trimester, it increases the rate of abortion or intrauterine fetal death of an infant [16].

Influenza virus

Influenza is a complicated virus causing major illness involving more than just a runny nose and sore throat, and the flu has a catastrophic effect when you are pregnant. Like others, flu is also transmitted by respiratory droplets from one person to another. The infectious period of flu may vary from day one to day seven [17]. 

The severe complication from flu to a pregnant woman is infectious respiratory diseases such as pneumonia. The fever from the influenza virus can give rise to numerous defects in infants. They are neural tube defects, structural changes after birth. These changes can alter the appearance and function of the newborn body. Many mothers who are in their first trimester and experiencing cold or flu along with fever are more likely to have a baby delivered with congenital disabilities. They are encephalocele, spina bifida, anencephaly, cleft lip with or without cleft palate, limb reduction, gastroschisis, bilateral renal agenesis, and colonic atresia [18].

When pregnant women did not suffer from fever, they are far less likely to encounter any defects in their newborn babies even though they had the flu. Being precautious and taking flu shots can help the baby to be born without any defects. This flu shot is safe for almost all pregnant women. The mothers who are allergic to eggs can get the flu shot under a medical practitioner's supervision as the shot contains an egg as a component. The other flu shot form can be nasal spray and is not recommended for pregnant mothers [18].

Varicella

Varicella-zoster is highly contagious for humans. The host for the Varicella virus is human. The transmission of the Varicella-zoster virus occurs through mucus membranes of the respiratory system and conjunctivae. The most common symptoms being the maculopapular rash with vesicles, which in turn cause itchy blisters. The infectious stage starts two days before the development of rashes. In a pregnant woman who is in their first trimester, Varicella can be launched by vertical transmission. The severity of Varicella can be seen when a pregnant woman is associated with congenital varicella syndrome, neonatal varicella, and maternal varicella pneumonia [19]. 

Clinical features of congenital varicella Infection are skin lesions, limb hypoplasia, microcephaly, hydrocephaly, cortical atrophy, mental retardation, microphthalmia, cataracts, chorioretinitis, muscular reduction, cardiovascular abnormalities, and learning difficulties. The Varicella-zoster virus is most likely to exhibit the above features only if pregnant women get chickenpox when 20 weeks or less. This is a sporadic infection for pregnant women and is called congenital varicella syndrome. Neonatal chickenpox transmits trans-placentally. If the mother gets infected before one to four weeks of delivery, then the newborn baby's infection percentage will be 50%. This is a life-threatening condition known as neonatal varicella. To avoid such effects, all the childbearing mothers should be vaccinated with varicella-zoster virus vaccination at least three months before conceiving. However, the mothers who got infected can also be saved by contacting their health care provider within 10 days of exposure [20].

Syphilis

Syphilis is a sexually transmitted disease. This infection can easily pass through the people having direct contact with a syphilitic sore called a chancre. The transmission can occur through vaginal, anal, or oral sex. The syphilitic sore forms after three weeks of direct contact and exhibits on the vagina, cervix, anus, rectum, and external genitals. They are usually asymptomatic in women and unperceived. The sore is small, round, painless, firm, and can last up to three to six weeks. These sores will give a pathway for HIV transmission by mucosal and epithelial barriers. If it is not diagnosed and treated during pregnancy, it can lead to serious illness to the fetus causing stillbirth, fetal loss, prematurity, low birth weight, neonatal and infant death, and many congenital diseases in newborns. With a lack of screening and proper treatment, 70% of all the infected pregnant women will face unpropitious results [21].

The spirochetes can cross the placental barrier to infect the fetus. From 14 weeks of gestation on, the infection worsens as the fetus's age increases. Congenital syphilis mainly depends on the stage of maternal infection, mother's treatment, gestational age, and immune response of the fetus. Infection to the placenta or the depletion of the fetus's blood flow is a frequent reason for fetal death. Clinical Manifestations of congenital syphilis include hepatomegaly, prolonged prothrombin time, jaundice, hematological manifestations, hydrops fetalis, anemia, mucocutaneous involvement (copper red maculopapular lesions, desquamation, rhinitis, saddle nose, condyloma lata), bone disorders, and nephrotic syndrome. An ample amount of maternal infection treatment would effectively prevent maternal transmission to the fetus and its treatment. The most common drug used to treat maternal syphilis is penicillin G [21].

Maternal Conditions

Diabetes Mellitus

Diabetes is nothing but a condition in which our body cannot satisfy enough insulin or when the body is totally immune to insulin in general. Insulin is a hormone in the human body. Insulin helps to get enough glucose into the body cells to be used as fuel whenever there is no external food source. In some conditions, glucose may not enter body cells, and the sugar stocks up in the blood leading to hyperglycemia [22].

The types of diabetes mellitus are (1) Type I: An autoimmune disorder where the body's immune system destroys the cells that make insulin in an organ called the pancreas, (2) Type II: This is a condition when the body is totally not capable of producing insulin at all. This is not an autoimmune disorder and (3) gestational diabetes, wherein blood glucose levels rise and various diabetic symptoms suddenly appear during pregnancy, as the same women may not be diagnosed as diabetic before. This is common for pregnant women [22]. In pregnant women, not only does their body makes hormones, but so does the placenta. The placenta is an organ that provides the baby with all the nutrients and oxygen needed to grow. The placenta makes hormones called estrogen, lactogen, and cortisol. All the hormones mentioned can block the insulin. This type of blockade is known as insulin resistance. It is insulin resistance that results in excess amounts of insulin in the mother's body and can create surplus sugar levels in the blood giving rise to gestational diabetes [23].

Risk factors of diabetes include obesity, women with a history of diabetes, inheritance from a family member who has type II diabetes, women with multiples or with twins, lack of physical activity, polycystic ovarian syndrome, previously given birth to a baby of weight greater than nine pounds. Women of Hispanic, Black, American Indian, or Asian American descent have a higher risk for gestational diabetes. Complications for the infant of a diabetic mother are stillbirth, birth anomalies mainly in the first trimester of pregnancy, macrosomia, birth injury, hypoglycemia, respiratory distress, and preeclampsia. Gestational diabetes can be treated assigning to your age, symptoms, general health. Gestational diabetes can be exposed during routine blood work in 24 weeks to 28 weeks of pregnancy. A medical practitioner can help glucose levels in the normal range throughout the pregnancy through small changes by incorporating healthy habits and foods to prevent gestational diabetes onset. Even though a woman had gestational diabetes before, the risk can also be lowered for the next pregnancy if they follow a healthy way of living and eating foods higher in fiber content, low fat, and calories. Devouring fruits, vegetables, and whole grains, along with watching the portion sizes, is recommended. Exercising daily for 30 min of a moderate workout can help you plan for your pregnancy at a healthy weight, not exceeding the recommended weight during pregnancy. All the above-mentioned factors will help to overcome gestational diabetes [23].

Epilepsy

Epilepsy is defined as a neurological disorder that involves recurrent seizures. Channelling of information from nerve cell to nerve cell occurs by an electrochemical process. When this process gets disturbed or follows a random pathway cause seizures. Around 25% - 40% of women with epilepsy suffering persistent seizures have more chances of getting them during pregnancy. The preferred medication for epilepsy may not work on pregnant women, as the absorption capacity alters during pregnancy. Some women experience nausea and vomiting throughout their pregnancy or at least in their first trimester. In this case of hyperemesis gravidarum, the medicine may not work, as it is emitted before explicating its complete effect [24].

Symptoms of epilepsy are headache, dizziness, fainting, memory loss, confusion, changes in energy level. Complications from Epilepsy during pregnancy include permanent brain damage, side effects from medications, injury from actions such as bumps, falls, self-biting, aspiration of fluid into lungs causing pneumonia, injury while driving or operating machinery, and learning difficulty, stillbirths, smaller babies, and pregnancy-induced hypertension. These are few complications that may encounter during pregnancy and after delivery but are not limited to the above points [25].

All pregnant ladies must take folic acid supplements 0.4 mg/day to prevent neural tube defects associated with the use of anticonvulsants by pregnant mothers. Some anticonvulsants affect vitamin D absorption. Most of the prenatal have vitamin D to conflict with this issue. Infant mothers who have epilepsy are at risk for imprudent bleeding. Few anticonvulsants affect vitamin K, which is the main factor for blood clotting. To prevent this, a newborn can receive an injection of vitamin K to prevent bleeding. A mother’s seizure medication can pass through breast milk in little amounts, but breastfeeding benefits outweigh these drugs' side effects [25].

Some anticonvulsant medications may be linked to birth anomalies. They are neural tube defects - valproate, carbamazepine; Intrauterine growth restriction - phenytoin, trimethadione; microcephaly - carbamazepine, trimethadione; low IQ - phenytoin, primidone, valproate; distal digital hypoplasia - phenytoin, phenobarbital, primidone; epicanthic fold - phenytoin, phenobarbital, valproate, carbamazepine, trimethadione; long philtrum - primidone, carbamazepine; hypertelorism - phenytoin, phenobarbital; developmental delay - phenobarbital, carbamazepine, trimethadione. Management of epilepsy during pregnancy can be done by close monitoring of the disorder and fetal health. More frequent visits to a specific medical practitioner can help control the disease and fetus [26].

Radiation

Radiation is usually considered as low risk in comparison with other risks of pregnancy. Almost for all the diagnostic radiation treatments and nuclear medicine examinations, pregnant women are exposed to less than 50 mSv. Such a low dose of radiation does not bring about any fetal health complications during pregnancy. Effects on fetal development include the cells in the embryo are proliferating, migrating, and differentiating at a higher rate; the embryo, which is rapidly developing, is extremely radiosensitive. The feedback after exposure to X- rays depends on the amount of dose given, dose rate, and radiation quality, the fetal stage at the time of radiation exposure [27].

According to the embryonic and fetal development stages, risk factors are significant during the early fetal stages and organogenesis. In the pre-implantation stage, the fetus undergoes either intrauterine death or resorption or normal fetal risk, when the threshold is 100 mSv. During organogenesis from the third to eighth week after conception, the risk rate decreases and may replace with congenital anomalies in developing organ systems. Growth retardation can occur if the pregnant woman is irradiated after four weeks of gestation with a possible threshold greater than 100 mSv. After eight weeks of gestation, the fetal anomalies risk factor decreases, whereas nervous system abnormalities, growth retardation, and childhood cancer can be more remarkable. Many of the abnormalities at the threshold of 100 mSv can be central nervous system-related [27].

Chemicals

Polychlorinated and Polybrominated Biphenyls

Polychlorinated biphenyls (PCBs) - The high levels of PCBs are detected frequently in persons working in industries that manufacture PCBs containing equipment. The PCBs are used as lubricants or coolants in most of the electric equipment. This occupational exposure to PCBs can be harmful to health and exhibits serious effects, such as greater liver enzymes, hepatic damage, dermal lesions, and respiratory problems [28].

Polybrominated Biphenyls (PBBs) - The PBBs are added to manufacture much plastic equipment like textiles, television, computers, and plastic foams. The manufactured item contains PBBs because they make them very difficult to burn. The degradation of PBBs is harmful to the environment. PBBs are the mixtures of brominated biphenyl compounds called congeners [29].

The PCBs and PBBs are the chemicals that can harm the fetus and the mother during pregnancy. These chemicals can cross the placental barrier in utero. The PBB shows its effects on fetal development, likely to include reduced fetal birth weight and a shortened gestational period. PCBs reveal their effects in utero associated with infants born with low birth weight and pre-term. Hence, vulnerability to PBB in utero is more than PCB in developing infants [30].

Syndromes

Fetal Alcohol Syndrome

Fetal alcohol syndrome is the effect of a mother's exposure to alcohol during pregnancy. With this condition, the child is at risk for brain damage and growth problems. The abnormality caused by fetal alcohol syndrome is not reversible. After the entry of alcohol into the bloodstream, it passes through the placenta and reaches the fetus. The fetus's alcohol is higher than the normal adult body because it metabolizes alcohol at a little rate than adults. In this way, the alcohol interrupts the oxygen delivery and nutrition to the developing baby. The effect of alcohol can damage tissue development and cause everlasting brain damage to the baby [31].

The signs and symptoms of a child experiencing fetal alcohol syndrome can be physical abnormalities, cognitive disabilities or intellectual disabilities, and functional problems associated with daily life activities. Physical defects include deformities of joints, limbs, fingers, vision difficulty, small brain size, head circumference, heart defects, kidney problems, bone defects, distinctive facial features, small eyes, thin upper lip, upturned nose, etc. [31].

## Conclusions

With frequent and proper medical guidance, pregnant women would not only gain awareness and understanding about teratogens, but they would be much more likely to prevent exposure during pregnancy. By learning how to identify teratogens and hamper them, expectant mothers greatly reduce the risk of congenital disabilities. The developing fetus is fragile, especially in early embryogenesis, when the body starts to build tissues and organs. Mindfulness and avoidance must start early, if not before conception. As more mothers learn about teratogens, it is imperative that society also becomes much more aware. For example, it is crucial that environmental agents, drugs, and chemicals used in the workplace or nature are to be tested to certify that they are non-teratogenic or should be averted to give to the pregnant women. Medical practitioners need to share alternate methods to alleviate aches, pain, tension, and viral illness in pregnancy to reduce risk factors of teratogens in fetal health during pregnancy through patient education. With improved efforts and reducing exposure, the resulting congenital disabilities would rapidly decline.
